# A platysomid occurrence from the Tournaisian of Nova Scotia

**DOI:** 10.1038/s41598-021-87027-y

**Published:** 2021-04-16

**Authors:** Conrad D. Wilson, Chris F. Mansky, Jason S. Anderson

**Affiliations:** 1 Department of Biological Sciences, University of Calgary, 2500 University Drive NW, Calgary, AB T2N 1N4 Canada; 2 Blue Beach Fossil Museum, 127 Blue Beach Road, Hantsport, NS B0P 1P0 Canada; 3 Department of Comparative Biology and Experimental Medicine, University of Calgary, 3330 Hospital Drive NW, Calgary, AB T2N 4N1 Canada

**Keywords:** Palaeontology, Palaeoecology, Zoology

## Abstract

The Hangenberg extinction has been hypothesized as a first order event in vertebrate evolution; however, information on the earliest Carboniferous vertebrate fauna, crucial in evaluating biodiversity changes, is scarce. Post-extinction recovery has been suggested as the driver of ray-finned fish (actinopterygian) richness increase and differentiation in the Carboniferous. Under this model, actinopterygian postcranial morphology differentiates in the second stage of their radiation. Here, we report on a platysomid occurrence from the Tournaisian of Nova Scotia, Canada. Despite long-standing taxonomic issues with deep-bodied actinopterygians, this specimen represents the earliest known occurrence of one such fish. Its presence in the earliest Carboniferous indicates that actinopterygians were already postcranially differentiated in the aftermath of the Hangenberg. Moreover, this specimen suggests that earliest Carboniferous actinopterygians used multiple locomotory modes; recent data from later Carboniferous taxa suggest that actinopterygian locomotory modes proliferated throughout the Carboniferous. Taken together, these data suggest that early Carboniferous actinopterygians were morphologically, ecologically, and functionally diverse.

## Introduction

Significant earth system changes occur during the Devonian-Carboniferous transition, including multiple extinction pulses^1^. The Frasnian/Famennian boundary pulse (the Kellwasser) constitutes ocean system turnover, including metazoan reef collapse^2^. The loss of ‘placoderm’ faunas and the Carboniferous diversification of actinopterygians and chondrichthyans has been linked to this transition (e.g. Long’s^3^ hypothesis of replacement). More recently, Sallan and Coates^4^ identified a significant shift in vertebrate clade-level faunal composition at the Devonian-Carboniferous boundary, which they hypothesized was driven by an independent mass extinction (the Hangenberg) selectively affecting gnathostomes.

These Earth system, ecosystem, and faunal composition changes appear to be accompanied by functional shifts in the global biota. Klug et al.^5^ and Whalen et al.^6^ revealed changes in water column occupation across global Devonian and Carboniferous faunas. Although nekton is common throughout the Palaeozoic, hydrodynamic animals with strong swimming capabilities (eunekton) first become proportionally more diverse than hydrodynamic animals without strong swimming capabilities (planktonekton) in the Silurian^6^. Changes in occurrences seem to occur later; when Whalen et al.^6^ analyzed data extracted from the Paleobiology Database, they found that eunekton proportional occurrences first exceed planktonekton proportional occurrences in the Carboniferous. This diversification and proliferation of hydrodynamic animals with strong swimming capabilities might be associated with adaptations improving swimming capabilities within locomotory modes, as well as the evolution of new locomotory modes.

Actinopterygians play a significant role in these changes. Actinopterygians appear depauperate in the Devonian, but become a major contributor to genus and species level diversity of the Carboniferous fauna identified by Sallan and Coates^4^. Thus, the timing of diversity, morphological, and functional changes in the actinopterygian fauna is critical in our understanding of broader faunal, ecosystem, and functional changes. For example, because the gnathostome clade-level faunal composition shift identified by Sallan and Coates^4^ occurs at the Devonian-Carboniferous boundary, they suggest that gnathostomes are selectively affected by the Hangenberg extinction, independent of extinctions earlier in the Devonian. Under this hypothesis, actinopterygians undergo an adaptive radiation in the Early Carboniferous when released from competition with other gnathostome groups^4,7^. Later, Sallan and Friedman^8^ interpreted changes in actinopterygian morphological disparity explicitly in this mass extinction context. In an empirical test of models of trait divergence derived from analysis of modern taxa, these authors found that the Carboniferous radiation was marked by the differentiation of cranial morphology and that post-cranial differentiation (primarily the exploration of a deep body-plan) occurred later^8^. Deep-bodied actinopterygians, as a component of Carboniferous actinopterygian diversity and a major axis of actinopterygian morphological^8^ (and associated functional) disparity are thus critical in understanding these changes and their timing.

**Figure 1:**
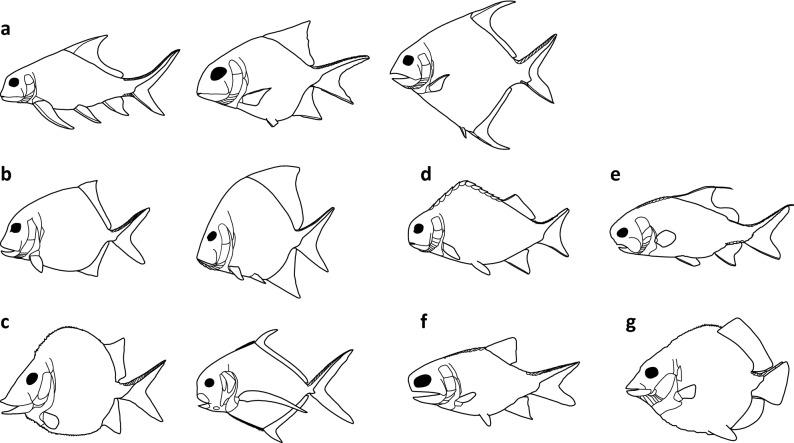
Diversity of body shapes among possible independent origins of deep-bodied actinopterygians listed by Sallan and Coates^9^. Lines on the leading margin of fins indicate fringing fulcra. (**a**) Eurynotiformes. From left to right: *Eurynotus*, modified from Sallan and Coates^9^, Fig. 15 and Traquair^10^, Plate III, Fig. 1; *Cheirodopsis*, modified from Sallan and Coates^9^, Fig. 15 and Moy-Thomas and Dyne^11^, Fig. 36; and *Amphicentrum*, modified from Sallan and Coates^9^, Fig. 15. (**b**) ‘Lower’ platysomids. From left to right: *Platysomus* *striatus*, modified from Haubold and Schaumberg^12^, Fig. 104; and *Platysomus* *superbus*, modified from Moy-Thomas and Dyne^11^, Fig. 39. (**c**) Bobasatraniiformes. From left to right: *Platysomus* *schultzei*, drawn from Zidek^13^, Figs. 22 and 24; and *Bobasatrania*, modified from Schaeffer and Mangus^14^, Fig. 11. (**d**) *Adroichthys*, modified from Gardiner^15^, Fig. 15. (**e**) *Aesopichthys*, modified from Poplin and Lund^16^, Fig. 12. (**f**) *Frederichthys*, modified from Coates^17^, Fig. 8. (**g**) *Discoserra*, drawn from Lund^18^, Figs. 5, 7, 8.

Unfortunately, Palaeozoic deep-bodied actinopterygians have been plagued by taxonomic and phylogenetic issues. Traquair^10^ grouped all deep-bodied Palaeozoic actinopterygians into the Platysomidae, which he concluded, in a typological argument, were specialized forms of the Palaeoniscidae. Later, Moy-Thomas^19^ divided Palaeozoic deep-bodied actinopterygians into two groups: the Amphicentridae and the Platysomidae. Whereas the interrelationships of deep-bodied actinopterygians have not been comprehensively tested through phylogenetic analysis, different small subsets of deep-bodied taxa have been included in some analyses (e.g.^20,21^). The results of these analyses vary; however, they agree that Palaeozoic deep-bodied actinopterygians are not monophyletic. Sallan and Coates^9^, after reviewing competing phylogenies of Palaeozoic deep-bodied actinopterygians, found as many as six independent Palaeozoic origins of deep-bodied actinopterygians: Eurynotiformes (Fig. 1a), Platysomidae–Bobasatraniiformes (Fig. 1b,c), *Adroichthys* (Fig. 1d), Aesopichthyidae (Fig. 1e), Frederichthys (Fig. 1f), and Guildayichthyiformes (Fig. 1g). This recognition of significant homoplasy among Palaeozoic deep-bodied actinopterygians has received support in recent phylogenetic analyses discussed by Friedman et al.^22^.

There appears to be at least one monophyletic group of deep-bodied actinopterygians in the early Carboniferous. Sallan and Coates^9^ proposed styracopterids (*Benedenius*, *Fouldenia*, and *Styracopterus*) as a sister group to amphicentrids, forming a monophyletic Eurynotiformes to the exclusion of all other Palaeozoic deep-bodied actinopterygians. A [*Styracopterus* + *Fouldenia* + *Amphicentrum*] clade has recurred in phylogenetic analyses^21,23,24^ and received support from further morphological investigation^22^, although this latest analysis treats *Benedenius* as an amphicentrid, not a styracopterid. These hypotheses focus on synapomorphic feeding structures^9,22^ in Eurynotiformes which, as in many other earliest Carboniferous actinopterygians, are durophagous^9,22^. Under this scheme, the eurynotiform body-plan deepens in the early Carboniferous—the late Tournaisian *Fouldenia* and the early Viséan *Styracopterus* are compressed relative to the deep-bodied late Viséan to Permian *Amphicentrum*. No eurynotiforms are known to survive the Permian–Triassic transition^9^.

However, our understanding of interrelationships between Palaeozoic deep-bodied actinopterygians, the relationships of these taxa to other actinopterygians, and the interrelationships of taxa within proposed clades remains generally poor. This is especially the case for *Platysomus*, the other putative Carboniferous origin^9^ of deep-bodied actinopterygians. This genus has needed revision for a long time^11,13,25,26^ because it is broadly inclusive of Palaeozoic deep-bodied actinopterygians. Because of this inclusiveness, *Platysomus*^11,26^ occurs through most of the Palaeozoic (Viséan—Lopingian). Although the genus *Platysomus* does not include post-Palaeozoic taxa, a putative platysomid-bobasatraniid lineage^13,27^ reaches the Mesozoic. Whereas the Viséan *Platysomus superbus* is the earliest described species of *Platysomus*^11^, the first *Platysomus* occurrence is controversial. Obruchev^28^ lists ‘*?Platysomus*’ among the early Tournaisian fish fauna of the Bystrianskaia Formation but includes no description, illustration, or specimen number. Zidek^13^ is skeptical of it and Sallan and Coates^4^ do not include it in their occurrences dataset. A better understanding of the first occurrence of platysomids (and deep-bodied actinopterygians more broadly) should help resolve the timing of key morphological, ecological, and functional changes in the Devonian-Carboniferous actinopterygian fauna. Here, we describe a platysomid specimen from the Tournaisian Horton Bluff Formation of Walton, Nova Scotia.

## Material and methods

### Institutional abbreviations

BWC, Barry W. Cameron Collection at the Blue Beach Fossil Museum, Hantsport, Nova Scotia, Canada; FMNH, Field Museum of Natural History, Chicago, USA; NSM, Nova Scotia Museum, Halifax, Nova Scotia, Canada; SM; Sedgwick Museum of Earth Sciences, Cambridge University, Cambridgeshire, England.

### Material

NSM 017.GF.017.001, Nova Scotia Museum of Natural History, Halifax, Canada, comprises an impression of the posterior of a deep-bodied actinopterygian, including the proximal parts of the dorsal, anal, and caudal fins. This specimen was listed and photographed by Mansky and Lucas^29^ in their review of the geology, ichnology, and palaeontology of Blue Beach, Nova Scotia, under a provisional specimen number, BWC 1029, but not described.

### Locality

**Figure 2:**
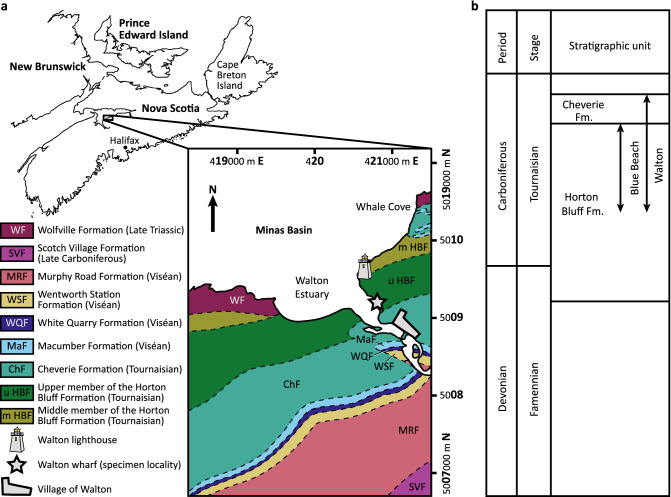
Geographic and stratigraphic context of specimen locality. (**a**) Geography and bedrock geology of Walton, Nova Scotia. Map of Nova Scotia modified from Anderson et al.^32^; map of Walton area modified from Moore et al.^30^. Numbers on map margins are UTM coordinates. (**b**) Schematic stratigraphic column of Blue Beach and Walton, Nova Scotia modified from Martel et al.^33^.

NSM 017.GF.017.001 was collected near the Walton wharf (Fig. 2a) by an unknown student and delivered to the late Dr. Barry W. Cameron at Acadia University. More recently, the specimen was passed to the Blue Beach Fossil Museum and received a provisional specimen number (BWC 1029) under agreement with the Nova Scotia Museum.

Exposures near Walton, Nova Scotia (Fig. 2a) are contained in the Kennetcook Basin. In the area of Walton, these exposures are members of the Tournaisian Horton Group (Fig. 2b); these are succeeded unconformably by Viséan members of the Windsor Group to the South. West-northwest and East-northeast of Walton, along the South margin of the Minas Basin, the Horton Group is contacted by the Triassic Wolfville Formation. Although the contemporaneous locality of Blue Beach (Fig. 2b) has yielded an abundance of vertebrate fossils^29^, Walton has not previously been known as a fossiliferous locality. Initial efforts to recover additional fossils from Walton wharf were unsuccessful. However, recent prospecting by one of us (C.F.M) below the headland, a short distance towards the Walton lighthouse from the Walton wharf (Fig. 2a), has yielded additional vertebrate material preserved in a large slab. As this slab was ex-situ, the specific strata and locality cannot be discussed with precision. Given the close proximity of this slab to the Walton wharf, it seems likely that NSM 017.GF.017.001 is from the upper member of the Horton Bluff Formation rather than the middle member of the Cheverie Formation.

The specimen is preserved in a highly indurated grey silty sandstone. The matrix does not exhibit any reaction to acid. The lithology of the specimen is consistent with the lithology of the Horton Bluff Formation^29^ and distinct from the red sandstones, evaporites, and carbonates of the Windsor Group and the brownish-red sandstones of the Wolfville Formation^30^. These Windsor Group and Wolfville Formation strata can be easily distinguished visually from the Horton Group in the Walton area.

Thus, the specimen is middle to late Tournaisian in age.

### Methods

**Figure 3:**
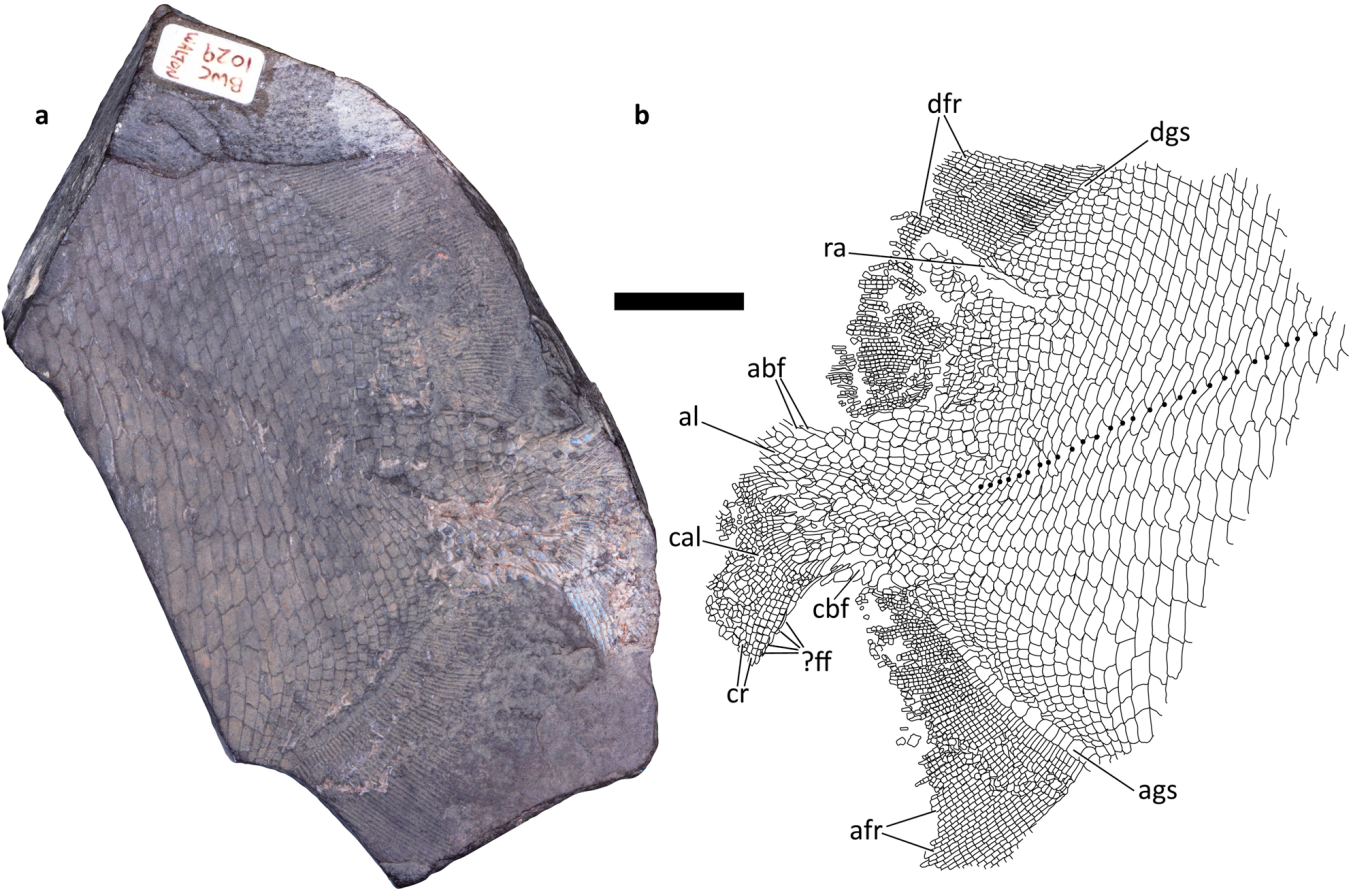
Whole specimen, including posterior flank and tail, of platysomid specimen NSM 017.GF.017.001. Scale bar = 20 mm. (**a**) Composite photograph of specimen in left lateral view. (**b**) Trace of latex peel taken from specimen—right lateral view of counterpart. Large dots indicate pores for the lateral line. afr, anal fin rays; ags, anal guard scales; abf, axial basal fulcrum; al, axial lobe; cal, caudal lobe; cbf, caudal basal fulcrum; cr, caudal lobe rays; dfr, dorsal fin rays; dgs, dorsal guard scales; ?ff, fringing fulcra; ra, dorsal fin radial.

C.D.W and J.S.A prepared a latex peel of NSM 017.GF.017.001 using Baird’s^31^ method. C.D.W photographed NSM 017.GF.017.001 and its peel with a Nikon D200 DSLR and a standard optical lens. C.D.W produced a composite high-resolution image of the specimen (Fig. 3) and the latex peel (Supplementary Figure S1) using the automate function in Adobe Photoshop CC 2018 (Adobe.com). Other figures were drawn using Adobe Illustrator CC 2018 (Adobe.com).

## Description

### Squamation

#### Flank scales

**Figure 4:**
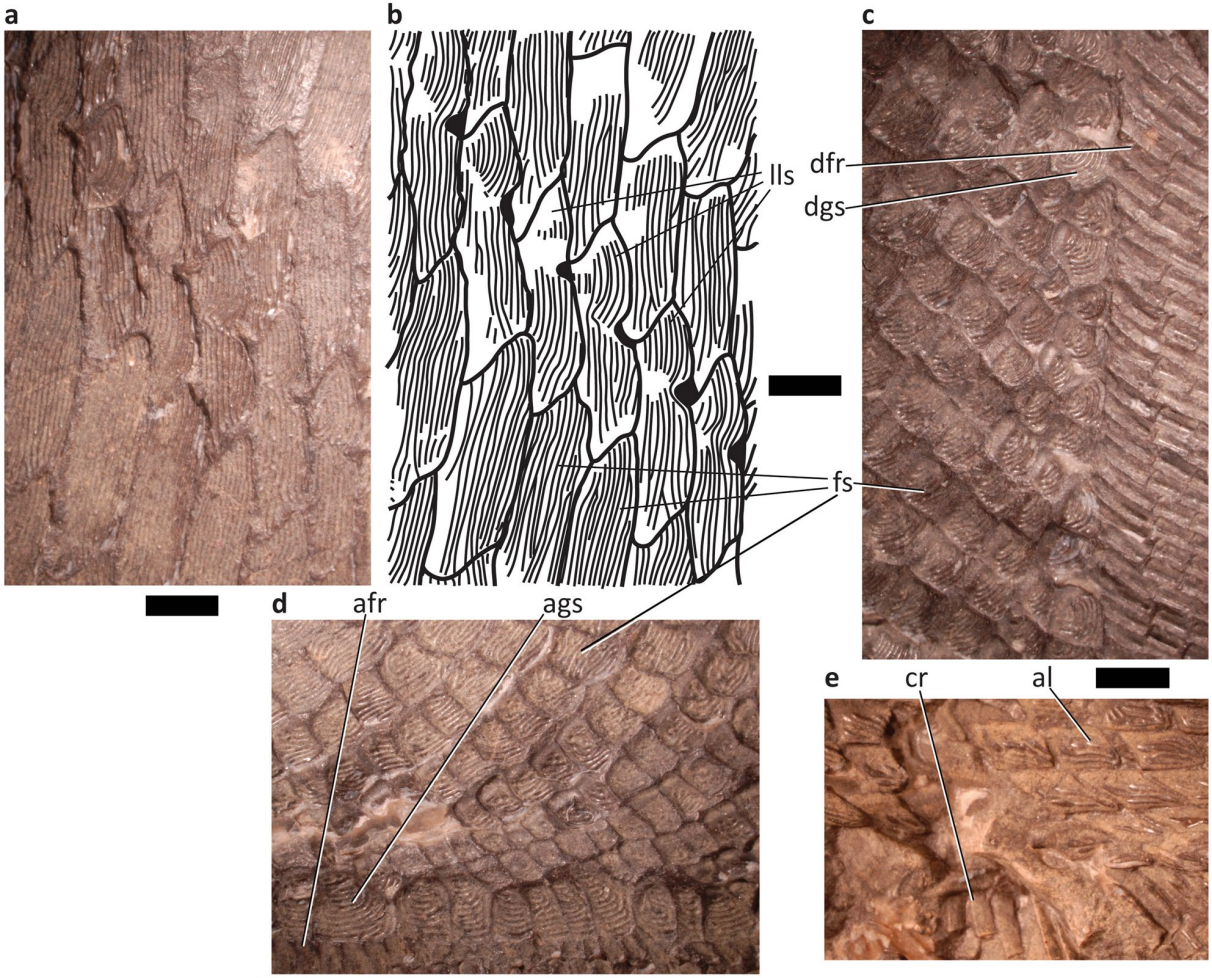
Detail of scale and proximal lepidotrichia of platysomid specimen NSM 017.GF.017.001. Scale bars = 2 mm. (**a**) Detail of flank and lateral line scales. (**b**) Line illustration of flank and lateral line scales. (**c**) Detail of scales and lepidotrichia proximal to dorsal fin. (**d**) Detail of scales and lepidotrichia proximal to anal fin. (**e**) Detail of scales and lepidotrichia in caudal fin. al, axial lobe; afr, anal fin rays; ags, anal guard scales; cr, caudal lobe rays; dfr, dorsal fin rays; dgs, dorsal guard scales; fs, flank scales; lls, lateral line scales.

At least 30 pre-caudal scale rows are preserved. The flank scales (fs, Fig. 4) are generally dorsoventrally deep and arranged in nearly vertical rows (Fig. 3). Dorsally, the rows are nearly straight and directed slightly anteriorly, and curve again anteriorly towards the ventral margin. The curvature is more pronounced in more posterior rows; the posterior-most rows are nearly sigmoidal. Scale size generally increases anteriorly and medially (Fig. 3). The largest scale impressions are subrectangular and tall with the longest margins parallel to the dorsoventral axis of the specimen. The scales become shorter towards the posterior margins, so the posterodorsal and posteroventral-most flank scales have their long axis perpendicular to the other flank scales. The dorsal and ventral margins of the imbricated scales are directed anterodorsally. These margins are also curved; the posterodorsal corner of the scales are pointed and convex, and the ventral margin of the next dorsal scale in the row curves around the posterodorsal corner of the previous ventral scale. The imbrication of the scales prevents examination for an anterodorsal process or peg and socket articulation. No keel is visible along the anterior margin.

Flank scales (fs, Fig. 4) bear seven to 15 fine ridges of ornamentation subparallel to the dorsoventral axis. The ornamentation of the small square scales (Fig. 4b–d) is deeply curved towards the anterior. In tall scales of the anterior flank, the posterior ridges of ornamentation undertake a forward-facing s-curve so that the ventral part of the ridge is anterior. Occasionally, curvature in successive ridges opens space where a new ridge is added; usually, the general decrease in ridge curvature in successive rows results in ridges in the middle of the scale pinching out. In some tall scales of the posteroventral quadrant of the flank, the pattern is reversed. Ridges along the posterior margin are straight and vertical, but some are interrupted as more anterior ridges curve posteriorly as they run ventrally. In some displaced scales in the posterior flank the dorsal and anterior margins form a flange devoid of ornamentation.

In lateral line scales, the ornamentation takes a similar but more drastic curve around the lateral line notch. Dorsal to the level of the notch, this forces the ornamentation to form concentric, forward-opening arcs that straighten out in successive rows towards the anterior, sometimes pinching out ridges in the middle of the dorsal part of the scale. Ventral to the lateral line notch, the ornamentation straightens and becomes vertical.

#### Guard scales

**Figure 5:**
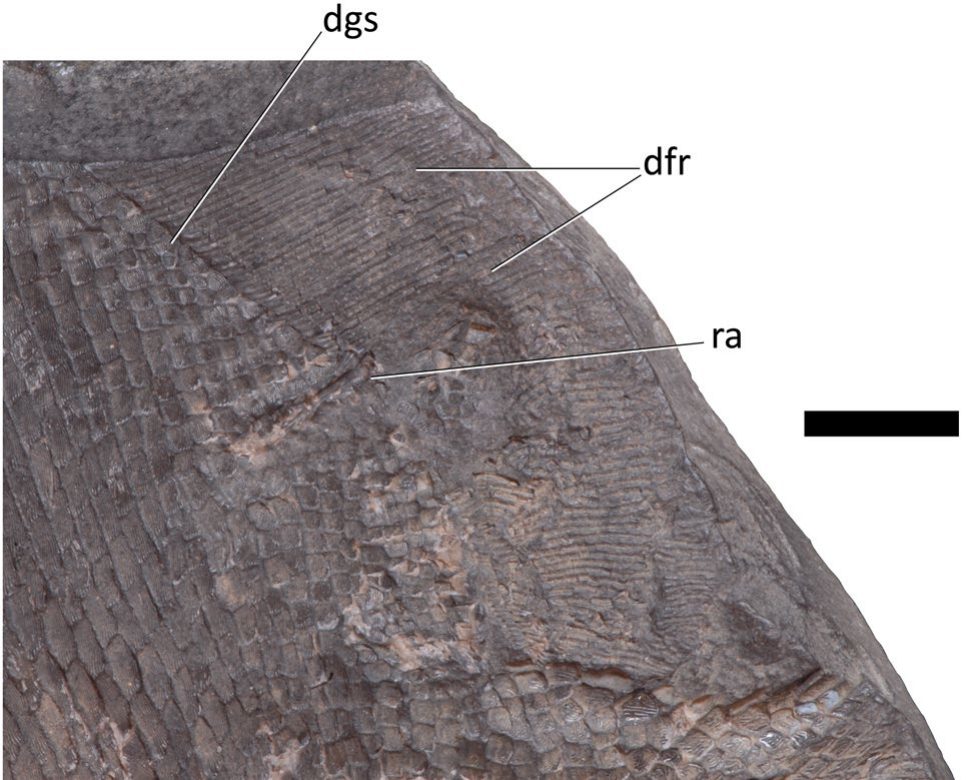
Detail of dorsal fin of platysomid specimen NSM 017.GF.017.001. Scale bar = 10 mm. dfr, dorsal fin rays; dgs, dorsal guard scales; ra, dorsal fin radial.

**Figure 6:**
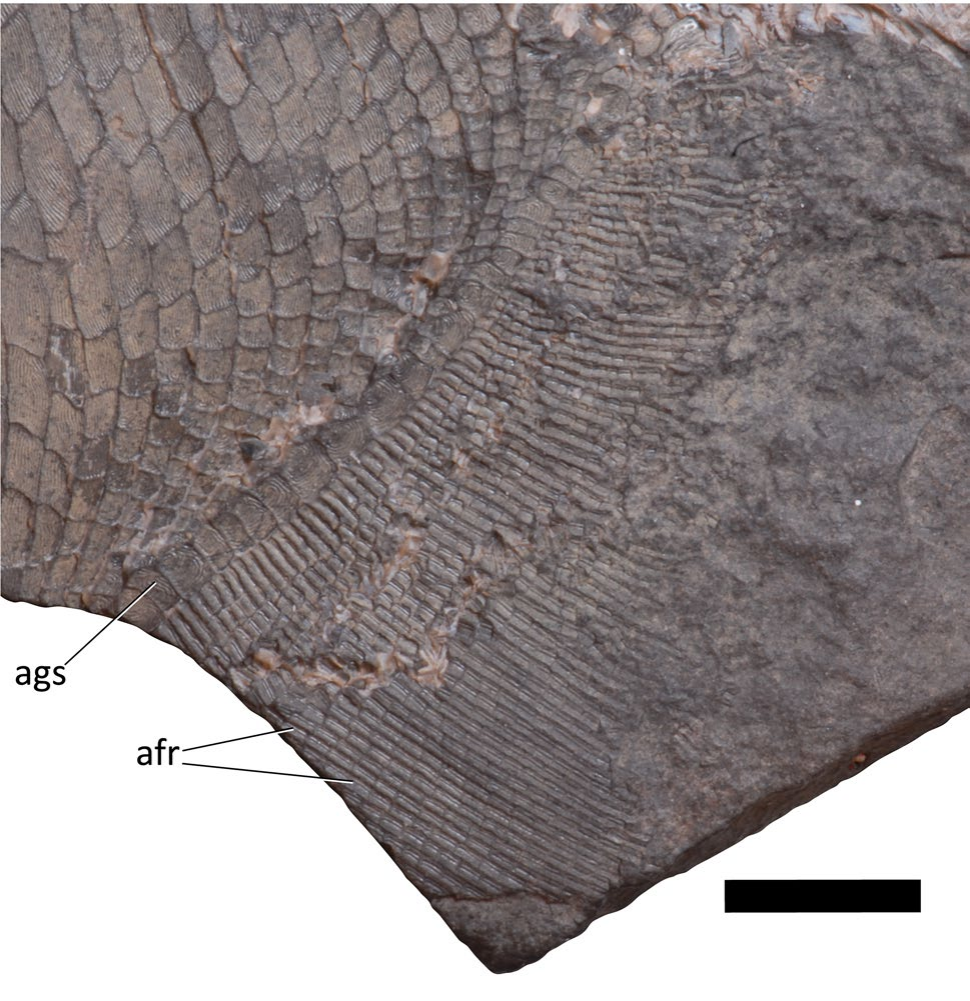
Detail of anal fin of platysomid specimen NSM 017.GF.017.001. Scale bar = 10 mm. afr, anal fin rays; ags, anal guard scales.

A single row of guard scales runs parallel to the base of the dorsal and anal fins (dgs and ags, Figs. 3, 4, 5 and 6), overprinting the pattern of curved scale rows in the flank. The dorsal guard scales (dgs, Figs. 3, 4 and 5) are slightly larger than nearby scales and bear curved ornamentation running subparallel to the ventral margin of the dorsal fin. Posterior ridges form concentric arcs, more anterior ridges are often straighter, and are occasionally truncated by the arc of posterior ridges. The anal guard scales (ags, Figs. 3, 4 and 6) are at least three times larger than adjacent scales and become larger anteriorly. The ornamentation of the anal guard scales is deeply curved and generally subparallel to the dorsal margin of the anal fin (Fig. 4c). Posterior ridges of the scale, if present, are briefly parallel to the posterior margin of the scale, then curve forward, following the ventral margin. These long posterior ridges are usually present in anterior anal guard scales and absent in posterior anal guard scales. Anterior ridges form tight forward opening arcs, small, nearly tuberculate ridges parallel to the dorsal and ventral margins, long s-shaped striae wrapping around multiple smaller ridges, or curve gently anteriorly as they run ventrally.

#### Scales of the peduncle and tail

The scales of the posterior flank grade into the scales of the peduncle (Fig. 5). Anteriorly, peduncle scales are rectangular, with ornamentation subparallel to the dorsoventral axis of the specimen. The scales become more elongate along the anteroposterior axis towards the posterior of the peduncle and are narrower towards the midline of the peduncle. Scales become rhombic in the axial lobe of the tail, with their longest axis parallel to the dorsal margin. These are compressed towards the midline and elongated posteriorly. Caudal scale row inversion appears to be abrupt, but the position of the hinge line cannot be established for its entire trajectory due to scale disarticulation. The axial lobe is seven scales deep.

**Figure 7:**
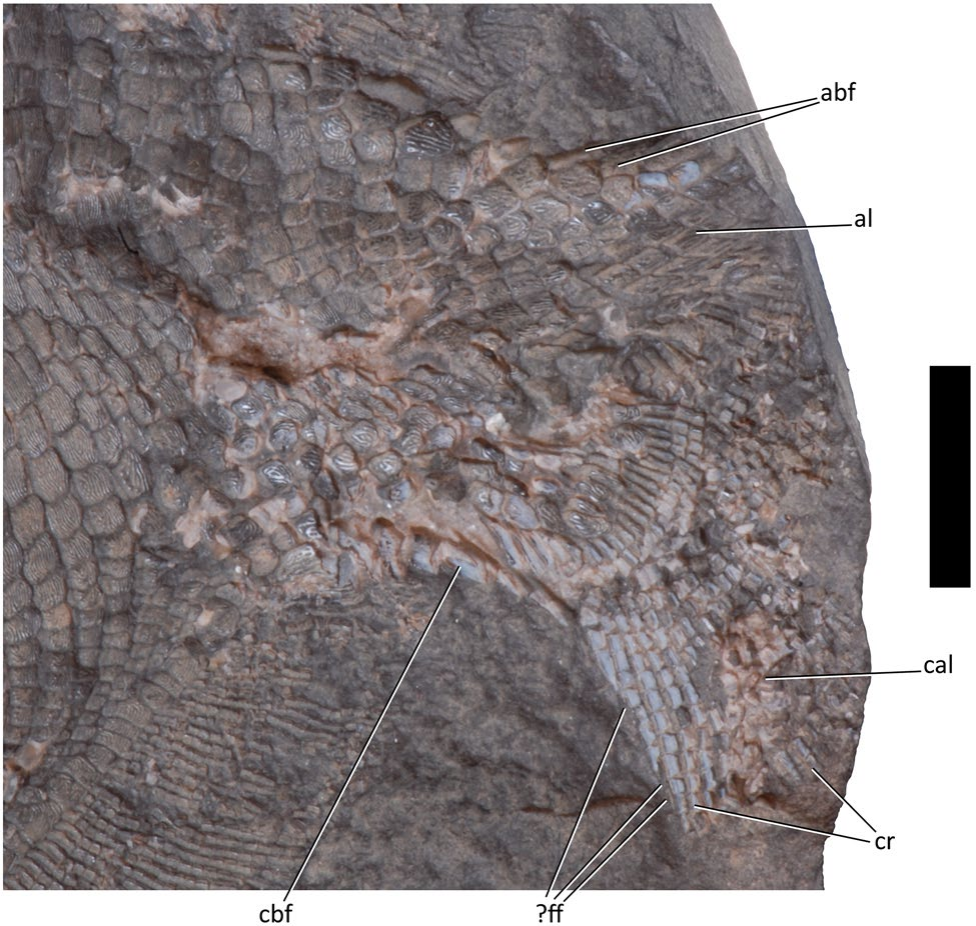
Detail of caudal fin and scales of platysomid specimen NSM 017.GF.017.001. Scale bar = 10 mm. abf, axial basal fulcrum; al, axial lobe; cal, caudal lobe; cbf, caudal basal fulcrum; cr, caudal lobe rays; ?ff, fringing fulcra.

Deeply imbricated axial basal fulcra (abf, Figs. 3 and 7) and caudal basal fulcra (cbf, Figs. 3 and 7) are present. The caudal basal fulcra (cbf, Figs. 3 and 7) originate at the posterior insertion of the anal fin. The insertion of the dorsal fin and the anterior-most axial basal fulcra (abf, Figs. 3 and 7) are poorly preserved. Both the axial and caudal fulcra are in a single row (abf and cbf, Figs. 3 and 7). The direction of scale ornamentation changes gradually in a posterior pointing V: it begins to run subparallel to the anteroposterior axis more anteriorly in scales dorsal and ventral to the caudal peduncle. Beyond the area of caudal scale row inversion, ornamentation ridges are usually larger and fewer. Diamond scales bear a few nearly tuberculate ridges following the longest axis of the scale. Ridges on rounder scales often form concentric arcs–ventral arcs open dorsally and dorsal arcs open ventrally. Both sets of arcs are often present, facing each other, but are sometimes replaced dorsally or ventrally by small straight ornamentations that fit within the arcs above or below.

#### Lateral line

Lateral line scales (lls, Fig. 4) bear a shallow notch in the dorsal-most third of the posterior margin of the scale. The lateral line runs dorsoanteriorly from the middle of the caudal peduncle, but its posterior fate is unclear.

### Median fins

#### Dorsal fin

The preserved dorsal fin includes about 72 fin rays (dfr, Figs. 3, 4 and 5), running from the dorsal margin of the specimen to the dorsal margin of the caudal peduncle. The dorsal fin cannot be evaluated for the presence of fringing fulcra or lepidotrichial bifurcation because it is incomplete distally. In the preserved extent, the fin rays are segmented up to 16 times without any evidence of bifurcation. As preserved, the posterior most ray contacts the first three axial basal fulcra; the dorsal and anal fin would have contacted or come close to contacting the peduncle in life.

The proximal most lepidotrichia are overlapped by dorsal guard scales (dgs, Figs. 3, 4 and 5); these are longer than their distal neighbours. Each dorsal guard scale overlaps 2–3 lepidotrichia. The lepidotrichia vary from rectangular to nearly square from the anterior to posterior of the fin margin. All well-preserved lepidotrichia bear a single median ridge.

A single dorsal fin radial (ra, Figs. 3 and 5) is displaced through the scale rows at the base of the dorsal fin. This is slender, but its distal end flares out. Broad, slightly elevated ridges running parallel to the left and right of the displaced radial may represent the position of other radials.

#### Anal fin

The preserved anal fin includes 56 fin rays (afr, Fig. 3, 4 and 6) in a series reaching the caudal peduncle. The dorsal most ray contacts the first three caudal basal fulcra (cbf, Figs. 3 and 7). The anal fin cannot be evaluated for the presence of fringing fulcra or lepidotrichial bifurcation because it is incomplete distally. The fin rays are segmented as many as 23 times without any evidence of bifurcation in the preserved extent.

The proximal-most lepidotrichia are overlapped by the anal guard scales (ags, Figs. 3, 4 and 6). Each anal guard scale (ags, Figs. 3, 4, and 6) overlaps 2–4 lepidotrichia. These are unsegmented and approximately twice the length of the next more distal lepidotrichium in the ray. Anteroventral lepidotrichia are rectangular and posteroventral lepidotrichia are nearly square. Lepidotrichia devoid of ornamentation, lepidotrichia with a single median ridge, and lepidotrichia with paired ridges on their posterior half are distributed irregularly.

In general, anteroventral lepidotrichia have more ridges than posterodorsal lepidotrichia, although the number of ridges varies within rays. The proximal-most lepidotrichia of the 30 dorsoposterior-most rays bear a single ridge of ornamentation, with lepidotrichia devoid of ornamentation occasionally present distally. The single ridge of ornamentation widens in proximal lepidotrichia anteroventrally, before splitting into paired ridges in the sixth proximal-most lepidotrichium of the 30th posterodorsal-most ray. Paired ridges are gradually found in more proximal lepidotrichia in more anteroventral rays; however, lepidotrichia with a single median ridge are still present in the anteroventral-most rays.

### Caudal fin

There are at least 34 fin rays in the preserved extent of the tail (cr, Figs. 3, 4 and 7). The caudal lobe (cal, Figs. 3 and 7) and axial lobe (al, Figs. 3, 4 and 7) are incomplete. Caudal lobe rays segment as many as 15 times without bifurcation in the preserved extent. Fringing fulcra may be present on the leading edge of the caudal fin (?ff, Figs. 3 and 7), but it is also possible that these structures are displaced hemitrichs from the opposite side of the fin. The proximal parts of the fin rays are unsegmented and six to seven times longer than their distal neighbours. The segmented lepidotrichia seem to become shorter in more posterior rays, so that lepidotrichia in the ventral part of the caudal lobe are often rectangular and lepidotrichia in the dorsal part of the caudal lobe are often square or shorter than they are wide.

Lepidotrichia in the ventral half of the caudal lobe bear a single median groove, parallel to the fin ray. More dorsal lepidotrichia, including the lepidotrichia of the axial lobe, may bear a single median ridge parallel to the fin ray or be devoid of ornamentation.

## Discussion

The characters of deep-bodied actinopterygians were recently reviewed by Sallan and Coates^9^. In previous analyses, character selection has emphasized the dermal skeleton and feeding structures, particularly the shape of the animal and its skull bones, and the dentition (e.g.^20^). This character selection seems to reflect the limitations of the fossil record, as many deep-bodied actinopterygians (including this specimen) are preserved in flattened lateral aspect. However, changes in body depth may drive broad, linked changes in morphology (e.g.^34^, Fig. 152–155), affecting the independence of these characters. Significantly, Sallan and Coates^9^ found that many characters used to evaluate the relationships of deep-bodied actinopterygians are homoplastic, dating back to Traquair’s^10^ typological argument that Platysomidae are specialized Palaeoniscidae.

The situation is especially dire for *Platysomus*. Zidek^13^ (page 167) summed up the situation: “… it is obvious that without a revision of all the noted species it cannot be decided what is and what is not a *Platysomus*.” Clearly, this statement only makes sense in context of deep-bodied actinopterygians—many things are verifiably not *Platysomus*. But there is very little, if any, distinction in meaning between *Platysomus* and “platysomid”. This is because platysomid genera cannot be distinguished from *Platysomus* (exemplified by Zidek’s^13^ transfer of *Schaefferichthys* to *Platysomus*) and because authors have continued to add species level diversity to *Platysomus*, noting the issues with the taxon, to avoid performing a genus-wide revision^13,26^. That this revision has been noted as necessary for more than a century e.g. (^11,25^) but has not yet been satisfactorily completed shows the difficulty and size of the problem. A further problem was created when Campbell and Phuoc^27^ united *Platysomus gibbosus*, the type species of *Platysomus*, with *Ebanaqua* and deep-bodied Triassic taxa in the Bobasatraniiformes without taxonomic revision. This carries the implication that at least two potentially distinct evolutionary lineages (Carboniferous platysomids and bobasatraniids) are present in *Platysomus*^27^. We note that although *Platysomus* and platysomids have generally been treated as monophyletic in analyses incorporating *Ebanaqua* (Zidek^13^ distinguishes between ‘higher’ and ‘lower’ platysomids within *Platysomus* and Sallan and Coates^9^ list Platysomidae–Bobasatraniiformes as a potential independent origin of deep-bodied actinopterygians), this monophyly of *Platysomus* has not been rigorously tested in phylogenetic analysis^26^. The relationships of species contained in *Platysomus* are unclear and platysomids, properly defined, may be inclusive of taxa that are not strongly deep-bodied (c.f. *Styracopterus*, *Fouldenia*, and the Eurynotiformes). Thus, here we take a similar approach to previous authors: we note the need for a revision of *Platysomus* and emphasize comparisons among and between platysomids sensu lato. Future revision of *Platysomus* and platysomids sensu lato may produce a clear, monophyletic definition of *Platysomus* and a platysomid clade, necessitating a stricter approach. In the interim and for the remainder of this manuscript, we use platysomid only in its broadest sense.

Given these general issues in character selection and the taxonomic issues surrounding *Platysomus*, it is not surprising that character selection for platysomids is also thorny. As noted above, many previously used characters (e.g. phyllodont dentition) are significantly homoplastic^9^. Some homoplastic characters are still useful operationally: Sallan and Coates^9^ contrast the heavy and highly imbricated fringing fulcra of eurynotiforms and the minute fringing fulcra of platysomids, which is useful when distinguishing between contemporaneous taxa. But minute fringing fulcra are also present in *Discoserra*^18^*,* so this is not an unambiguously platysomid character even among deep-bodied actinopterygians.

Scale morphology and ornamentation appears significant. Sallan and Coates^9^ distinguish between the central scale pegs of eurynotiforms and the anterodorsally placed pegs of *Platysomus* (closer to the condition in fusiform non-neopterygian actinopterygians). All members of *Platysomus* share a scale ornamentation that is usually described as fine, vertical striations^9^. However, this ornamentation can be more complex. In NSM 017.GF.017.001 specimen (as is figured for *Platysomus striatus*^35^, Plate 17), the ornamentation is slightly curved, pinching out some striae and opening space for others. This is similar to the condition of anterior scale ornamentation observed in some species of *Mesopoma* (i.e. *M. smithsoni* and *M. pancheni*) in which anterior striae curve posteriorly as they run ventrally, although the posterior scale ornamentation is distinct^17^.

Characters distinguishing the multiple lineages within *Platysomus* are better established but can also be problematic. Campbell and Phuoc^27^ proposed 15 characters supporting their platysomid-bobasatraniid lineage, but Zidek^13^ noted that many cannot be used because they are widespread among actinopterygians or because alternative states cannot be distinguished. Zidek^13^ added several characters, but some also have problems: we cannot distinguish the parasagittal rows of caudal fulcra proposed as diagnostic for the platysomid-bobasatraniid from the pairs of caudal fulcra arranged in chevrons that are widespread in non-neopterygian actinopterygians^36^ in the data given.

Because the overall shape body shape of NSM 017.GF.017.001 is most similar to members of *Platysomus* (Fig. 1b,c) and the scales bear distinctive sub-linear ridges of ornamentation (Fig. 4a,b), the specimen appears to be a platysomid. Unfortunately, characters of the skull and scales that might be useful in placing this specimen are not preserved because of specimen breakage and scale imbrication, respectively. Similarly, specimen preservation precludes evaluation of the characters defined by Campbell and Phuoc^27^ and Zidek^13^ for the platysomid-bobasatraniid lineage ('higher' platysomids). No described platysomid-bobasatraniids have fringing fulcra on the leading edge of the caudal lobe, so the putative fringing fulcra in this specimen may be a point of difference.

**Figure 8:**
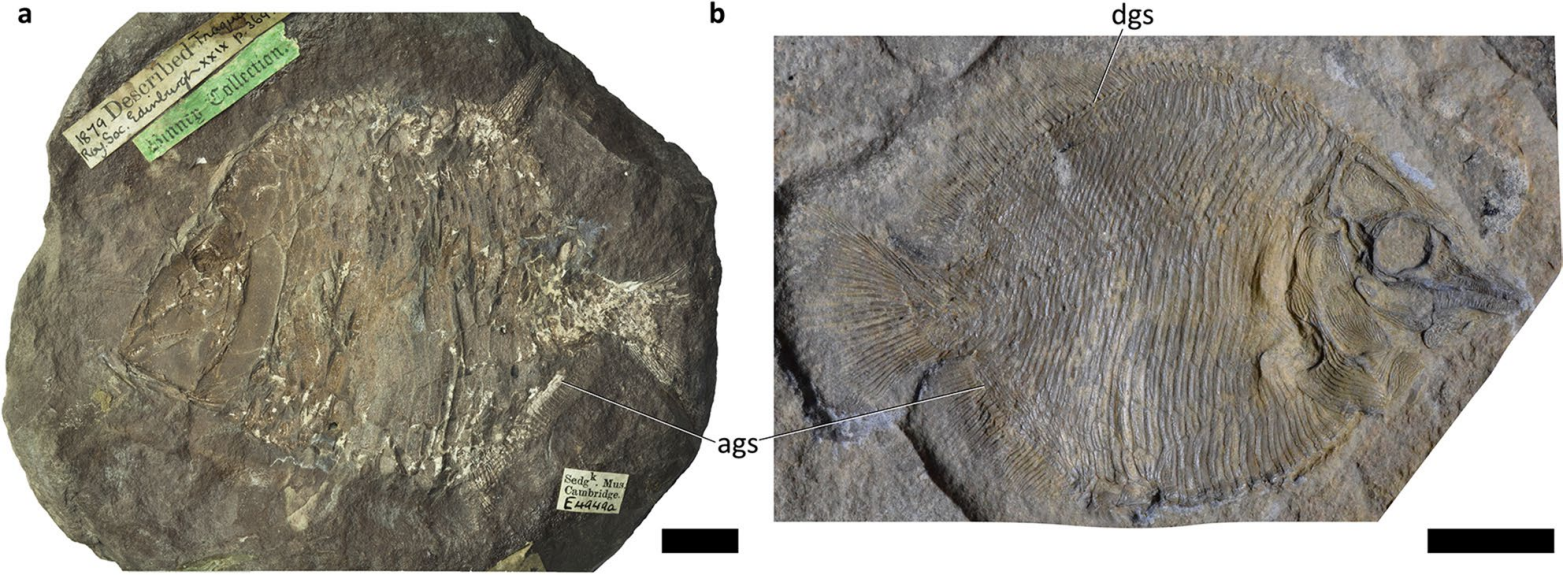
Other platysomids bearing anal and/or dorsal guard scales. Scale bars = 10 mm. (**a**) *Platysomus tenuistriatus*, SM E 4949 a in left lateral view. Photograph provided by the GB3D Type Fossils Online project, the copyright holder, at http://www.3d-fossils.ac.uk/fossilType.cfm?typSampleId=20005040 under a Creative Commons Attribution-NonCommercial-ShareAlike 3.0 Unported License. (**b**) *Platysomus* sp., FMNH PF 10792 in right lateral view. Photograph kindly provided by Jack Stack. ags, anal guard scales; dgs, dorsal guard scales.

More significantly, dorsal and anal guard scales are present in this specimen. Some specimens of *Platysomus tenuistriatus* (SM E4949 a and SM E4949b, Supplementary Table S1) also have anal guard scales (Fig. 8a, ags) and an undescribed platysomid from Bear Gulch, FMNH PF 10,792 has both dorsal and anal guard scales (Fig. 8b, dgs and ags). This allows for comparison with other deep-bodied taxa, although this comparison is not comprehensive. Guard scales have not been described before in deep-bodied actinopterygians, despite their presence in *Platysomus tenuistriatus*, and may be underreported. Dorsal and anal guard scales are also present in *Lineagruan* spp.^20^; however, this specimen differs from *Lineagruan* spp. in body depth and the absence of pectinate posterior scale margins.

The extensive differences between these platysomid specimens include the guard scales. Whereas 2–3 lepidotrichia contact each dorsal guard scale and 2–4 lepidotrichia contact each anal guard scale in NSM 017.GF.017.001, the ratio of guard scales to lepidotrichia is approximately 1:1 in *Platysomus tenuistriatus* and 1:2 in FMNH PF 10792. Nevertheless, the presence of dorsal and anal guard scales unites NSM 017.GF.017.001 and some members of *Platysomus*, reinforcing this specimen’s identity as a platysomid. Furthermore, *Platysomus tenuistriatus* and FMNH PF 10792 are from the Viséan and Serpukhovian, respectively, so NSM 017.GF.017.001 appears more similar to close contemporaries than later Carboniferous platysomids.

The presence of a platysomid in the Tournaisian has consequences for our understanding of actinopterygian diversification and differentiation. This specimen likely predates the late Tournaisian *Fouldenia*, the oldest known eurynotiform taxon^9^, making it the earliest described deep-bodied actinopterygian. Although no morphometric analysis has been performed here, this early deep-bodied actinopterygian would seem to expand the morphospace occupation of Tournaisian actinopterygians along the post-cranial axis in the dataset of Sallan and Friedman^8^ and weaken the interpretation that cranial disparity increases before post-cranial disparity in Palaeozoic actinopterygians.

Locomotory strategies are important in this context of actinopterygian post-cranial disparity. Many of the wide range of locomotory strategies used by modern actinopterygians—including both body and/or caudal fin (BCF) locomotion and median and/or paired fin (MPF) locomotion—are first employed by Carboniferous actinopterygians. The elongate body-plan of Carboniferous tarasiids^37^ evinces anguilliform (eel-like) locomotion^11^, an additional BCF locomotory mode. Fusiform non-neopterygian actinopterygian locomotion was long assessed as similar to chondrosteans^38,39^, as these taxa seem to share negatively buoyant armature, a heterocercal tail, and limited pectoral fin flexibility. Nevertheless, Coates and Tietjen^39^ recently revealed a flexible “rowing” pectoral fin in *Trawdenia planti*, which indicates that pectoral fin based MPF locomotory modes were present in at least one fusiform Carboniferous actinopterygian.

Early Carboniferous deep-bodied actinopterygians also imply new locomotory strategies, since a laterally compressed, deep body is associated with unsteady swimming and low straight-line speed but high maneuverability^40,41^. Furcacaudiform thelodonts have been suggested as Devonian explorers of a deep body-plan^41^, and the Devonian acanthodians *Brochoadmones*^42^, *Ptomacanthus*^43^, and *Cassidiceps*^44^ have been described as deep-bodied, moderately deep-bodied, and relatively deep-bodied respectively. However, ‘deep-bodied’ is a subjective descriptor that encompasses a wide range of anatomy, and clearly these animals explore a deep body-plan in a different way from actinopterygians. These taxa lack the long, posteriorly placed dorsal and anal fins which are ubiquitous in deep-bodied actinopterygians (although we note the confluence of the anal and caudal fins in *Brochoadmones*^42^), and which modern deep-bodied actinopterygians undulate to effect ballistiform locomotion^40,41^. Although these modern taxa are highly specialized, the placement and flexibility of the anal and dorsal fins of *Ebanaqua*^27^ and the bulky musculature of these fins interpreted in the description of *Discoserra*^18^ suggest that Palaeozoic deep-bodied actinopterygians used some form of this gait in conjunction with other fins. Depending on the configuration of the pectoral fins and buoyancy^39^, the pectoral fins might be used for locomotion and fine-positional control^27^.

The deep body-plan and the configuration of the dorsal and anal fin in NSM 017.GF.017.001 is a strong signal indicating a more maneuverable locomotory strategy relative to fusiform contemporaries^40,41^. This does not imply the full exploration of a ballistiform gait, but the long dorsal and anal fins in the specimen and observed modifications for dorsal and anal fin locomotion in other Palaeozoic deep-bodied taxa^18,27^ suggest that this MPF locomotory mode was used to some extent. Although the pectoral fins of this specimen are not preserved, the analysis of Coates and Tietjen^39^ indicates that pectoral fin based modes of locomotion exist in Palaeozoic actinopterygians. Thus, the number of locomotory modes known for actinopterygians increases from one (a form of BCF locomotion) by the Famennian to three (BCF locomotion and MPF locomotion with the anal/dorsal fin and the pectoral fins) by the Tournaisian at the latest, and to at least five (with pectoral fin rowing in *Trawdenia* and anguilliform locomotion in tarasiids) later in the Carboniferous. This could support the hypothesis that the post-Devonian actinopterygian radiation encompasses locomotory differentiation. Since tight correlations between changes in feeding and locomotory structures have been previously observed in Actinopterygii^8,45^, one possibility is that locomotory changes opened new feeding opportunities in deep-bodied lineages during their invasion of new niches and ecospace as part of broader turnover among (especially durophagous^7^) vertebrate predators^4^, congruent with the more general ‘feeding-first’ model considered by Sallan and Friedman^8^. This specimen and other Carboniferous taxa showing ecological and locomotory differentiation provide only a minimum age for these changes. Analysis of Devonian (especially Famennian) actinopterygian locomotory and feeding structures will be critical in evaluating the feeding-first hypothesis and understanding actinopterygian response to ecosystem change.

This occurrence might also provide an additional minimum age for actinopterygian diversification. When *Platysomus* spp. are included in phylogenetic analyses, they tend to be recovered as deeply nested in a radiation of post-Devonian actinopterygians (e.g.^20,21,23,24^). Indeed, Giles et al.^21^ recover *Platysomus superbus* as the stratigraphically oldest member of the actinopterygian crown, so its occurrence (~ 334 Myr) provides the minimum age of the actinopterygian crown in their fossil tree. This is younger than their preferred molecular tree, which places the appearance of the actinopterygian crown at 359.9 Myr. If the phylogenetic placement of *Platysomus superbus* in this analysis reflects platysomids more generally (*Platysomus superbus* is usually considered a non-bobasatraniid/‘lower’ platysomid^13^), then the recovery of this Tournaisian platysomid helps resolve this discrepancy between the molecular and fossil tree. This supports the hypothesis that the appearance of the actinopterygian crown occurs later^21^ than previously thought and is congruent with the view that a major actinopterygian radiation starts in the Early Carboniferous, post-Hangenberg^4,21,46^. However, it remains possible that critical divergences occur earlier and diversification starts pre-Hangenberg^23^.

Understanding the actinopterygian radiation requires evaluating diversification models (e.g. the explosive, long fuse, and short fuse models of Archibald and Deutschman^47^), but this is difficult without a better understanding of actinopterygian interrelationships. The observation that an actinopterygian showing morphological and functional disparity is present by the Tournaisian at the latest might be helpful in this context. Genus-wide revision of *Platysomus* and phylogenetic analysis of deep-bodied actinopterygians in context of other Palaeozoic actinopterygians might further illuminate these problems.

## Supplementary Information

Supplementary Information.

## Data Availability

All data generated or analyzed during this study are included in this published article and its Supplementary Information files.
