# Supplementary material for: A platysomid occurrence from the Tournaisian of Nova Scotia
Source: Sci Rep. 2021 Apr 16;11:8375. doi: 10.1038/s41598-021-87027-y (PMC8052371; doi:10.1038/s41598-021-87027-y)
Supplement: Supplementary file 1 — Supplementary Information. [file 41598_2021_87027_MOESM1_ESM.pdf]

Supplementary information for: A platysomid occurrence from the Tournaisian of Nova Scotia

Conrad D. Wilson, Chris F. Mansky, and Jason S. Anderson

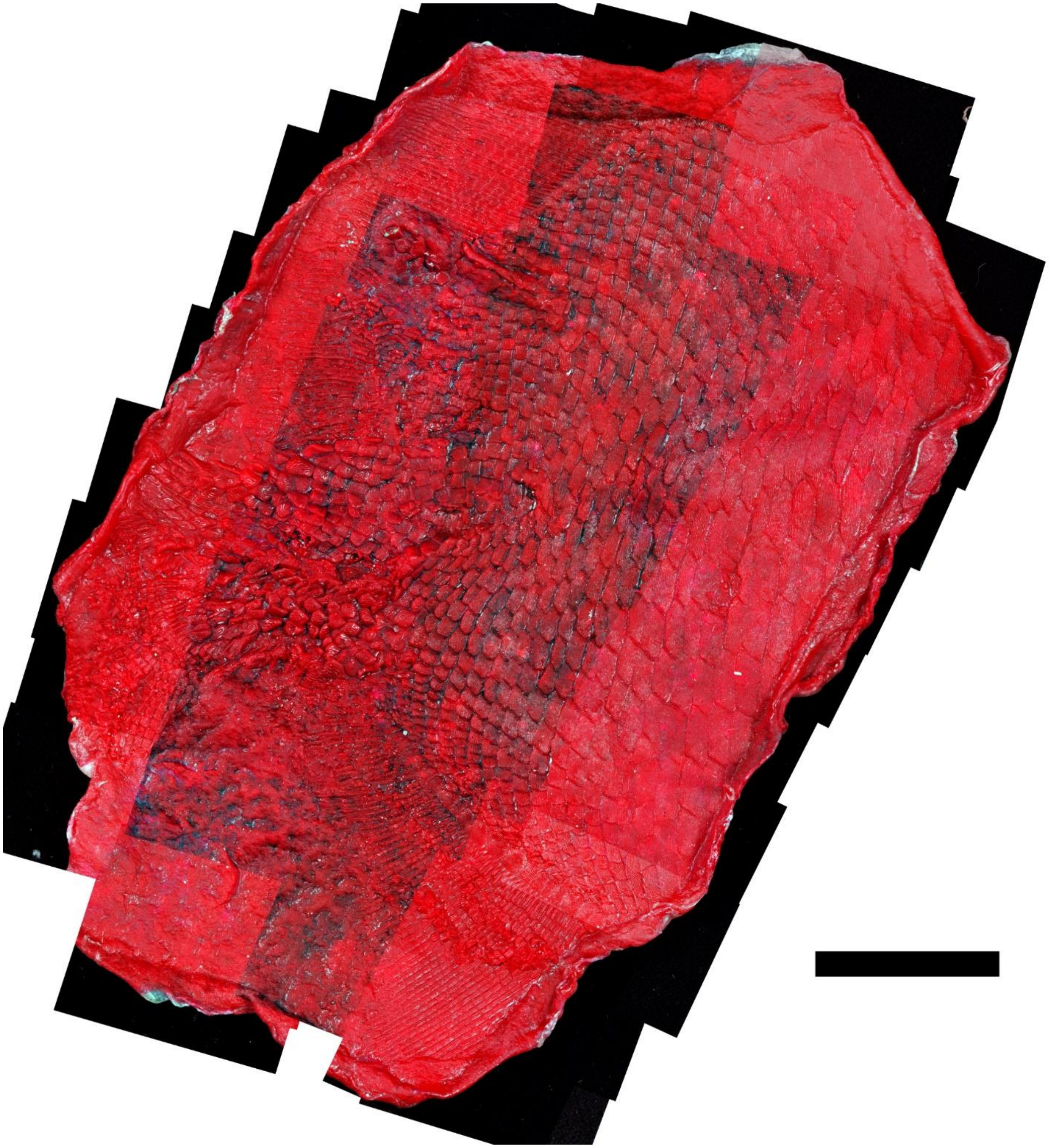

Supplementary Figure 1. High-resolution composite image of latex peel created from platysomid specimen NSM 017.GF.017.001. Scale bar = 20 mm. Peel was used with direct observation of specimen for analysis and for the creation of manuscript Figure 3.

Supplementary Table 1. British *Platysomus tenuistriatus* specimens bearing guard scales with links to high-resolution photographs.

Specimen Identification	Photograph
Sedgwick Museum E4949 a	http://www.3d-fossils.ac.uk/fossilType.cfm?typSampleId=20005040
Sedgwick Museum E4949 b	http://www.3d-fossils.ac.uk/fossilType.cfm?typSampleId=20005041
